# Chapter III: Typhoid Fever

**Published:** 1883

**Authors:** 


### CHAPTER III.

### TYPHOID FEVER.

The modern literature of typhoid fever is not scanty; the sum of patient observation devoted to the disease by modern physicians has been somewhat commensurate with the importance of the subject; the results gathered have settled much that but recently was requisite to an intelligent understanding of the subject. Its history, etiology, pathology, symptomatology, diagnosis, and prognosis have been ably treated, and the works of Louis, Bretonneau, Andral, Chomel, Cloquet, Cruveilhier, and others, among the French; and Schœnlein, Rokitansky, Skoda, Virchow, Canstatt, Griesinger, and many others, among the Germans; Graves, of Ireland; Smith, of England; and Bartlett, of our own country, have left but little to be desired in these elements of a complete knowledge of the disease. So much of intelligent thought and labor could not but yield its fruits, and if these have not been all that a benevolent hope might have expected, they have at least been such as all can rejoice in, so far as they have contributed to establish the truth of this most important subject. They should not be undervalued by any student however plainly he may see, after passing over their careful handling of these elements, that these laborers and writers have seemed to stop just where he could have wished them to have gone on, *i. e.*, to the discovery and establishment of a system of cure equally worthy of their genius, and his respect and confidence. As to the elements named, they have left little to the domain of doubt; as to a system of cure, they have left little else. In the present paper, therefore, we propose to accept these elements as they have been presented by received authorities, only protesting against what is believed to be a great error in the department of pathology which stands directly in the way of all improvement in the therapeutics of the disease. We refer to that teaching which puts effects in the place of causes; which regards the *products* of morbid action as the disease itself; and views local deposits and changes of tissue as the sum of the evil with which we have to do, instead of considering them, as it should, only as the *partial results* of that sum of the modified action of the vital forces which alone constitute the disease. To regard the peculiar state of Peyer's patches, so generally found in dissections after death from typhoid fever, as the disease itself, is scarcely more wise or philosophic than to exalt the sordes on the teeth or the cracks on the tongue or lips to this dignity. All are alike merely results of the state which for convenience we have so named. If the science of pathology is to either aid or control our therapeutics (it has been declared its province to do both by some), then a view so restricted and erroneous as that here alluded to must be followed by disaster to the progress of therapeutics toward the perfection hoped-for, and for which all profess to strive. A partial pathology cannot be followed by perfection in therapeutics—nor a false pathology by safety in practice. Hence the fact, otherwise surprising though true, that the mortality of this fever has been scarcely if at all diminished by the combined labors of all these great lights in medical science. They have given much labor, observation, and thought to the subject, and the result of all has been the fact that certain changes are usually wrought in Peyer's patches during the progress of the fever, and this has been abundantly proved by autopsies enough to prove anything they are capable of proving, and the other fact, that a peculiar eruption appears on the skin of patients, supposed to be significant of this fever and peculiar to its second stage. Beyond these two facts it is not certain that any addition has been made to the knowledge of its pathology possessed by their predecessors, and these are not material helps to the discovery of a system of successful therapeutics. It is not quite easy to perceive how such a system could be discovered without previous reference to the law of cure, and the existence of such a law they all ignored. The only possible result followed: their patients and the patients of their followers continued to die.

Homœopathy claims to have discovered a better system—a system founded on law—on law which the Almighty made a part of man's being when he created the first of the race and made him subject to disease. It claims for this system a better success than can come from any other not founded on law; it promises to all who apply it in practice a success just equal to the precision of the compliance with the requirements of this law, and promises success to no others.

To the "doers of the law" only is it pledged; not to those who only swear by its name. The application of this law to the treatment of typhoid fever is the object of the present paper.

The first duty of the physician in attempting this treatment, is a *thorough examination of all* the elements of his case. This cannot be too strongly insisted on or too carefully performed. In no other disease is this so important, though it is indifferent in none. Having honestly discharged this duty, and ascertained not only that he has the fever to combat, but all the elements it now reveals, let him consider them well and carefully before he decides on his first prescription, and be *positively sure* that it is *right*, before he ventures on the first dose, for no subsequent effort in the case is of equal importance. If the first prescription be wrong, no subsequent pains may be sufficient to remedy the consequences of the blunder. A confusion of the case from this source has often been realized which no skill could remove. If the *first* prescription be right all the subsequent course is comparatively easy. If wrong, there is only vexation, difficulty, and anxiety before the physician, and to the patient and his friends, it is too likely, there is only a certain fearful looking for of pain, danger, and death. Let it never be forgotten that *time* here is of no account, if the question be of time or a wrong prescription. Let whatever of time the case may require for accuracy be given to it, no matter to what extent, for it is infinitely better for the patient that we do nothing than that we do wrong. When the younger Bœnninghausen won his great and perhaps unparalleled success in the treatment of this fever with a single remedy, he recognized and practiced this principle. He made sure of the accuracy of his *first* prescription, and then he fully recognized and obeyed the second essential rule of successful practice, viz.:

Having found the right remedy, keep to it—*change it for no other* but for the *strongest reason*. Let no impatience for more speedy results, nor that honest and honorable desire to do better, which all right-minded practitioners must feel and which is likely to be especially active with the young, tempt to do wrong by an unwise change of remedy. If the first be right, to change it for another will most likely be to give one less appropriate, and the result will too often cause bitter regret. The writer was years in learning the importance of this rule of practice, and the memory of the experiences from which it was derived is mixed with much that is painful, which he has now no doubt a better practice might have avoided.

In treating typhoid fever it should be constantly borne in mind that it is not often that the disease can be crushed at the first blow. It may require patient and protracted watching and care before the looked-for amendment appears, but however long these may have been, the *time* furnishes no reason for substituting another remedy for that in use, this having been selected because its pathogenesis was most similar to the characteristic elements of the case. And now, whatever the time of its administration may have been, it is only to give place to a successor for a similar reason, greater resemblance to the elements of the case. Examples of both acute and chronic diseases are constantly met in practice which require medication for a longer or shorter period, according to the nature of the given case, before amendment becomes apparent, though the prescription may have been most accurate. Such cases are more frequent among typhoid fevers than other acute diseases. They call for the most careful revision of prescriptions, but never for a change of remedy except for the reason given above. Success in such cases only follows a steady adherence to that which has thus been ascertained to be the right. When, as sometimes happens in this fever, this period of what may be called latent medication is protracted days or even weeks, and the course recommended requires more than common firmness and the exhibition of much of that quality called *nerve*, there can be no doubt but that he who has not these qualities, or is unwilling to use them, is not fit for the responsible duty of treating such cases.

And further, if emulous of the best success, having selected the best remedy, we are not to be tempted into giving another less appropriate with it, expecting thereby to add anything to the curative effect of the principal remedy, or by this resort to excite a second and independent curative process by the power of the supplemental remedy, and so by this to add to the sum of the curative effect which can be realized in a given time. This too common resort of *alternating remedies*, as it is called, is rarely admissible in the treatment of this fever, though it is feared it is not seldom practiced. So far is it from being necessary or in any way beneficial, as a rule, the writer has no hesitation in declaring it positively mischievous. The practice has with many become a habit, with so many, indeed, that it may be regarded as a fashion. Entered upon in the first instance with no very definite idea of principle involved in it, and repeated without reflection, repetitions come almost as a matter of course in every prescription, for whatever disease. Against this as a general mode of practice we protest, it being without the support of any principle, uncalled for by any necessity of an intelligent practice, and, as often witnessed, is nothing else than a violation of that most necessary and wholesome law of our school which limits prescriptions to a single drug at a time. The giving of two in succession at the short intervals which are too common, is in nothing better, so far as a compliance with this law is concerned, than giving both from the same glass at the same time. Against this as a practice in the fever under consideration we protest, as against a method almost always extremely pernicious and not unfrequently fatal. The exceptions are so few that it certainly can and ought to be entirely discarded. It is certain that this view is not in accordance with the current practice, and therefore not with the current judgment of the day. But it is confidently believed to be true, notwithstanding. That its truth is not more generally perceived is explained by the power of habit, and perhaps in some cases of prejudice. Let us look at the matter a moment:

What is the reason for giving any remedy in any disease? What but the resemblance of its pathogenesis to the elements of the case under treatment? This resemblance is found and the remedy is given, and is it not equal to the demands of the case? The promulgation of the homœopathic law was accompanied by the declaration of its sufficiency. Has intelligent experience falsified that declaration? If so the whole of Homœopathy falls to the ground, and its advocates are no better than self-deceivers or the deceivers of others, and its practitioners are under a delusion more dark and dangerous than that which gives confidence to the advocates of the school they oppose. If the one drug so selected be sufficient, then the second is in no way called for. If it be not equal to the cure, then it is submitted that this fact is a call rather for a revision of the prescription than for the administration of a second drug in connection with it, probably no better chosen. But it may be said, and often has been, that cases occur where there is great difficulty in finding the required resemblance between the drug and the disease. This is true, but what then? The practice of Homœopathy is not easy. It never has been. It was never adapted to the demands of the lovers of ease. Such persons have nothing to do with it. If they *honestly* attempt to have, it will be only a trouble to them. But do persons who plead the above difficulty as an excuse for the practice under consideration pretend that the difficulty is lessened by the duty of finding *two* similars instead of *one?* Or is it that before the difficulty, it being so great, they feel justified in abandoning allegiance to the homœopathic law, and in attempting to accomplish by *two* remedies which are dissimilar to the disease what the law declares can only be accomplished by *one* which is similar? Can two remedies which are not in curative relation to the disease, *i. e.*, are dissimilar, do more good than one which is similar? Is there in all that is known of disease and drug action, and of the law of curing, any reason for believing that such a practice can be in the slightest degree beneficial? It is confidently believed that it is generally entered upon without thought and that it is wholly unprofitable.

There is another *habit* of practice, too freely indulged, against which in this connection it is our duty also to protest, because in the treatment of this fever it has been often followed, and ever will be, by the gravest mischief. The allusion is to giving Aconite without discrimination to all cases of acute attack where there is a hot skin, thirst, restlessness, and a quick pulse. To give Aconite as a matter of course, where there are only these elements of fever, is no better than sheer empiricism, and has for its defense no better reason than the current popular notion that *Aconite is good for fever*, which, if by it be meant that it is good for all cases of fever, is only, like many other popular notions, merely a popular falsehood. It is only "good" for fevers which present symptoms like its pathogenesis, which typhoid does not. In the early stage of typhoid, it is just the remedy to do more mischief, when out of place, than any other. This follows from the inevitable law of drug action. The state of the patient is, with the rarest exceptions, just the opposite of that to which Aconite is appropriate, and therefore the drug is antipathic, and consequently intensifies the evil it was given to control. This objection to Aconite applies with most force to its use in cases which occur in dense and crowded populations, or which have resulted from whatever protracted exposure to influences and circumstances that depress the vital forces. The fever to which Aconite is related is one of exalted action, never of depressed. Indeed, it is nearly limited to cases based on *local irritations or inflammations*, with tendency to *fibrinous deposit*. In these cases even its usefulness is confined to the stage of deposit, or that of irritation, which immediately precedes it. For example, in pneumonia, after the deposit of fibrin, Aconite is no longer of the least service. It has been given in such cases a thousand times, because there was still high fever, and Aconite is *par excellence* the remedy for this, but a thousand times it has here disappointed us all, because of our failure to appreciate the relationship here asserted.

It is our duty also to protest against giving Bryonia or Rhus tox. in the early stage, or in any stage of this fever, merely because it is typhoid, and because these remedies chanced to be once in the curative relation to an epidemic of historic celebrity. This, of course, can be no reason for continuing their use in epidemics to which they sustain no such relation, or in sporadic cases for any other reason than that their pathogenesis is more like the symptoms of the case to be treated than the pathogenesis of any other drugs. Above all, we protest against their ever being given, as they have been too often, in alternation, for this, in addition to the reasons given above, that the conditions to which the one is appropriate is just the opposite to that of the other, and never the same.

The foregoing practical rules, it will be observed, are part positive—care in the *first* examination and analysis of a case, precision in the *first* prescription, and adherence to this remedy when once decided on—and part negative. We are not to alternate remedies, and we are to avoid Acon. in the first stage, with very rare exceptions (no one but a madman would think of giving it in a subsequent stage), and are neither to give nor alternative Bry. and Rhus because it is typhoid fever. With these rules before us, having complied with the first requirement, how shall we proceed to attain the exactitude required by the second ? In the first place, we are to discriminate between symptoms which are of controlling importance in the decision of the question of remedy and those which are not. How ? By taking chiefiy into consideration those symptoms which are indicative of a peculiar condition, and giving to those which are of less significance a secondary place in the account. Thus, the peculiar character of the patient's mind and disposition, as to delirium, stupidity, irascibility, etc., are far more significant of the condition of the brain than mere pain in the head, however severe ; and the characteristics of the intestinal evacuations than pains in those organs. The pains are common to all cases—are generic—the peculiarities above specified are those which individualize the case, and therefore are its characteristics ; it is not to be forgotten that these are here and always the decisive symptoms in the selection of remedies.

We will suppose the case to be in the first stage of the attack, what symptoms shall we be likely to meet? We will present, presently, the group given by Prof. Griesinger, of Tubingen, in Virchow's *Handbuck der Speciellen Pathologie und Therapie*, 2te Band, 2te Abtheil., s. 130, as one of the latest and best pictures from the writers of the old school of the phenomena of this stage. We do this, not because it is perfect for the purpose of a homœopathic prescription, but as, when taken in connection with translated symptoms of our Materia Medica, furnishing a convenient opportunity to answer, in part, the often-repeated declaration of opponents of our school, that the phenomena of diseased action do not find a parallel in those of drug action. In this statement of the symptoms of the first stage of typhoid fever the author had no reference to any record of our Materia Medica, he simply intended to give a representative picture of the disease; the parallel symptoms of the Materia Medica were on the record long before Griesinger wrote. There was, therefore, no collusion. It is given also because of its *representative character*, *i. e.*, it presents the symptoms the most important, as the author understands them; and the most frequently met in his practice, *i. e.*, belonging to the greatest number of cases, thus furnishing a convenient opportunity, not only to present an answer to an oft-repeated and groundless objection, but also the proper method of dealing with a record of symptoms where a prescription is to be made. It also demonstrates the truth of the principle, already stated, when having to speak of Acon., that remedies related to depressed vital force are those from which we shall find our curative, even in this first stage. Of these Ars. is certainly one of the chief. It is rare that a record of symptoms in practice can afford an opportunity for a more perfect compliance with the demands of the law of cure than these of Griesinger, when met by Ars.*

But cases in practice are not constituted according to the models of the text-books. The symptoms of each case, while presenting generalities sufficiently like these, show also special symptoms or combinations by which it is characterized and which constitute it an individuality. And in this individuality the case must be studied, and to this the remedy adapted by the law of similars. Typhoid fever, in its earliest existence, chiefly attacks and modifies

* For the parallel of Griesinger and Ars. see the symptoms of Ars. and Griesinger to be given hereafter.

the functions of the brain and of the organs of the digestive apparatus. In its early stage these modifications are the proper objects of most careful study. It is in the functions of these organs that the fever, for the most part, makes its existence known, and in these it is to be combated. In the study of cases at the bedside, it will be found, that early in the history of the attack a preponderance of important symptoms has shown itself in one or the other of these spheres, which marks the first step in the analysis of the case, and that which decides whether the remedy is to be found in the class of drugs which attack the brain by preference, of which Belladonna may, for convenience, be received as the representative; or in the class which by similar preference attacks the digestive apparatus, represented by Arsenicum; or if the case be mixed, *i. e.*, the two systems about equally affected, in the class of drugs equally related to both, of which Bryonia may stand as a type. Where cases are marked with strong cerebral or abdominal preponderance, this classification may help somewhat to find the right remedy. But as cases in practice will not make themselves up in models for our convenience, it is to be understood that in this analysis we only contemplate *preponderance*, all cases being more or less mixed in their cerebral and abdominal manifestations.

In this paper we propose to examine the relations of Bell., Hyos., Lach., Opium, and Stram. to typhoid fevers with predominant cerebral symptoms. After the symptoms have been presented in groups it will be easy for the student to perceive the resemblances and differences of the different groups, and from these differences to decide on the selection of either which may be appropriate to his case.

*Belladonna*. Trembling with sense of weariness in the limbs (in the early stage). Heaviness and weariness in the limbs; great debility, and general weakness with sleepiness in the afternoon; exalted irritability of all the organs; congestive tendency of the blood to the head; red spots like flea bites, or like blood stains, or petechiæ, on the chest, abdomen, face, and neck.* Great drowsiness; profound comatose sleep with snoring (see Opium); sometimes opening the eyes, without moving, with wild look. Jerking of the tendons, with pale face and cold hands, and small, hard, rapid

* If this reminds the homœopathist of the "lenticular rose spots" of Louis, he is not therefore to exalt the importance of this symptom into decisive authority in the selection of this drug, because this writer made it a pathognomonic of this fever.

pulse. Sleeplessness with strong desire to sleep; sudden waking from sleep with start and fright; sighing and jerks in sleep which wake the patient; frightful visions on closing the eyes to sleep; anxious and frightful dreams; shudderings from the slightest current of air; face red, swollen, with injected conjunctiva. Furious delirium with violent pain in the forehead. The patient suddenly springs from the bed, or attempts to. He is timid and fearful and suspicious, and desires to run away (in a later stage); reluctant to answer questions or to speak; increased sensibility of all the senses; is quarrelsome, and strikes, bites, and spits on his attendants. The patient sings, laughs, and talks loud in his delirium. He is completely insensible as if in a dream, he neither sees nor hears. Illusions of the senses and imagination; he has visions of beauty or terror; the delirium is violent with staring of the eyes; it is also loquacious, or perhaps muttering. (The violent and loud is more frequent with this drug.) He talks of dogs, wolves, cattle, soldiers, battles, and of going home. Vertigo with anxiety, and glimmering before the eyes; throbbing of the carotid and temporal arteries, and also in the forehead. The pains in the head are increased by the movement of the eyes; the eyes are prominent, red, staring, sparkling, brilliant, distorted, or affected by spasmodic motions. The pupils are either contracted or greatly dilated and immovable, or the eyes may be dull and without expression. Deafness; burning heat and redness of the face; distortion of the mouth; dry and cracked lips, or they are dark red and dry; dryness of the mouth and throat extending from the fauces into the nose. Tongue red, hot, dry, and cracked; it is red on the margin and white in the centre; trembling of the tongue; paralytic weakness of the organs of speech (see Lach.); heaviness of the tongue; difficult stammering speech, as if drunk; difficult speech from difficulty of respiration, and great debility. The above are the symptoms of Belladonna which bring it into relation to typhoid fever. It will be at once seen that for the most part they are referable to a modified state of the brain, and that the drug is by them related to both the early and later stage of the disease.

*Hyoscyamus.*—Jerking of the limbs and tendons; debility and weariness of the whole body; uncommon sinking of strength; universal debility with trembling of the whole body and coldness of the extremities; burning of the skin while laying the hand on any part of the body. Uncontrollable disposition to sleep; continued profound sleep; gentle sleep; quiet sleep with profuse perspiration; coma vigil; sleeplessness from nervous excitability; perspiration in sleep; suffocating snoring with the inspiration; carphologia; waking with outcries; anxious dreams; burning heat, both external and internal. Pulse small and weak; small and thready; rapid and intermittent; very small, hardly perceptible; weak and irregular. Sweating with great weakness and stupidity; insensible stupidity, which is conscious of no want except thirst. Entire loss of consciousness, and of the functions of the organs of the senses; does not recognize relatives or friends; illusions of the imagination and senses. Delirium with the fever, which is continued while awake, and which sees persons who are not and have not been present. Indistinct and muttering loquacity; muttering with picking of the bed-clothes; inability to think, the thoughts cannot be directed or controlled; vacant staring at surrounding objects, with apparent entire self-forgetfulness. Vertigo, like drunkenness. Dull pain at the base of the brain, in the forehead, and especially in the membranes of the brain. Heaviness of the head, with sense of empty confusion, and severe pain. Stupefying pressure in the forehead, which passes into shootings or tearings (on the left side). Eyes sparkling and red; prominent and convulsed; distorted and staring; staring at surrounding objects; weak, dull, without lustre; squinting; pupils either much contracted or dilated; deafness; offensive smell from the mouth; tongue red, hard, dry, and clean or coated brown. Burning dryness of the tongue and lips, which look like burnt leather. Paralysis of the tongue. Embarrassed, indistinct speech; answers no questions; loss of speech and all sensibility; distention of the abdomen with pain on pressure; tympanitic distention; watery diarrhœa; involuntary and unnoticed stools in bed. Paralysis of the sphincter ani; suppressed secretion of urine; retention of urine; involuntary urination; paralysis of the bladder.

*Lachesis.*—Painful weariness of the limbs, extending from the elbows and knees; relaxation of the muscles, with exhaustion from the slightest exertion. Weakness, with inclination to sleep; dullness and bruised pains in the limbs; trembling of the limbs, and internal trembling, with fever and faintness, evenings. Sleepiness with weakness in all the limbs; great dullness of mind with bodily weakness; complete insensibility; delirium at night; much muttering during the evening fever; heaviness of the head, with dullness, like lead in the occiput, with vertigo. Throbbing in the head from every movement; congestion to the head; heat in the head. Eyes weak and dull or distorted; sensibility to light; deafness; very sensitive to sound; rushing and thunderings in the ears; bleeding from the nose; hanging of the lower jaw during the coma; dryness of the mouth, with thirst or constant desire to drink. Dryness of the tongue; black; as if stiff, with difficult motion while swallowing; paralysis with difficult protrusion of the tongue; difficult speech with heaviness of the tongue; nasal, indistinct speech; pain in the epigastrium on pressure; distention of the abdomen, also hard, with gurgling and rumbling in the bowels before the diarrhœa.

*Opium.*—Unconquerable weariness, or weariness like intoxication; uneasiness with sense of illness of both body and mind; heaviness of the limbs; weakness, with aversion to all external objects, persons, and things, with drowsiness, silly stupidity, sadness, and weakened memory. Great prostration and depression of spirits; weakness, with inability for any work; marked sinking of strength, with inability to move himself, with want of tone in the solids of the body. Fainting and vertigo with every attempt to leave the bed; emaciation of the body; small, red, itching spots, here and there on the body; coma vigil, with indistinct muttering. Stupefying slumber also at night, with increased thirst; tongue almost clean, with hard, bright red edges, and cracked lips. Irresistible sleep and complete coma, with insensibility; heat, pulse and respiration natural. Constant slumber with carphologia, and touching surrounding objects, with half-opened eyes. Profound sleep, with loose, rattling respiration. Stupid sleep with unconsciousness; stupid, comatose sleep, with stertorous breathing; half-open mouth; distorted, open eyes; red, puffy face; hanging of the lower jaw; respiration slow, heavy, or even intermittent; pulse slow or suppressed; jerkings of the limbs and muscles of the face and corners of the mouth; sleeplessness, with restlessness and delirium, or with incomplete visions and phantasies. Impossibility to sleep, though feeling very sleepy; nocturnal alternation of coma vigil and coma somnolentum, with delirium, hot skin, and stupidity; stupid sleeplessness with phantasies of dragons, skeletons, horrible spirits, ghosts, in a state of half-sleeping and waking. After morbid sleep, stammering; difficulty of moving the tongue; nausea; anxious and frightful dreams.

In stupefying sleep, stertor, especially during *expiration;* whimpering, sighing, and moaning; suffocating nightmare; pulse first rapid and strong, then weak and intermitting; rapid and weak, with rapid, oppressed, and anxious respiration; copious perspiration, with itching miliary eruption, with insensibility of the organs of touch, sight, and smell. He sits in silence, absorbed in himself; loss of consciousness; dullness of the senses, with heavy eyes and extreme weakness; perfect loss of consciousness and insensibility with relaxation of the muscles. He neither knows his relatives nor the most familiar objects; dullness and stupidity of the intellect and all the senses; stupid insensibility to both pain and pleasure; imbecility; stupidity of the senses, with watery eyes; anxious respiration, with strong heaving of the chest; slow comprehension of ideas; internal dullness, as if sleepy and drunk; sensibility entirely benumbed. Delirium; visions; frightful phantasies of mice, scorpions, etc., with desire to run away. Delirious talk of ghost, devils, spirits, which he says surround his bed and afflict him. Delirious muttering of old occurrences, with open eyes, and recognizes what is said to him only as if after a dream; furious delirium. In his delirious fancies the patient does not believe he is in his own house. Heaviness of the head; great heaviness of the occiput, like lead, so that the head constantly falls backward. Inability to hold up the head; throbbing of the arteries of the brain; congestion of the head; eyes open and turned upward; staring and unusually bright; glassy, prominent, immovable, expressionless, like one dying. Stares on surrounding objects, with watery eyes, without comprehending what occurs or recognizing his relatives; pupils contracted, dilated, or immovable; insensibility of the iris to light; ringing and rushing sounds in the ears; stupid aspect, with relaxed and hanging facial muscles and lower lip; distended nostrils, and difficult raising of the upper eyelids; distortion of the mouth; convulsive trembling of the facial muscles, lips, and tongue. Tongue dry, without thirst, especially in the morning; black tongue; paralysis of the tongue; difficult speech, can only speak loud with great effort. Stammering; distension of the abdomen; hard, with tension and pain from pressure. Tympanitis; diarrhœa, extremely offensive or watery; involuntary stools; retention of urine, as if from a closing of the bladder or a loss of its power. Respiration slow, deep-drawn, and sighing; stertorous, deep-drawn, with loose rattling or loud and difficult; interrupted intermittent, with moaning.

*Stramonium.*—Trembling of the whole body, or of one or more of the limbs; weariness of the limbs, great weakness, prostration, strong inclination to lie down; can only walk a few steps without support; red miliary spots on the chest and back; small, shining, star-shaped petechiæ on the face, throat, and chest. Profound sleep with stertorous breathing, or with very deep-drawn respiration with great effort; coma, with loose rattling respiration, with dark, brown face; waking with loud outcries; pulse weak, irregular, often intermittent, small, rapid, or hardly perceptible, very small, rapid and intermittent, imperceptible. He is fearful and excited; desires to run away; believes he is always alone and is terrified; strikes his attendants, with fearful outcries; great disposition to bite and tear everything with his teeth, even his own limbs; sudden alternations of laughing, singing, and weeping. Loss of consciousness; imbecility; stupefaction of the senses; he takes no notice of what occurs; neither sees nor hears nor recognizes his relatives; insensible to external impressions; speaks to the absent as if they were present, and calls inanimate objects by the name of persons, while he takes no notice of his attendants. Illusions, as if his body were cut into in the middle; as if all surrounding objects were very, very small, while he himself is very large and elevated on high; believes he sees a great company of people about him, and grasps at them. Frightful objects are constantly before the imagination while his expression is that of fear or terror; believes he sees dogs, cats, rabbits, approaching him from all around, and that he sees ghosts. Delirium; loquacious; mild; terrified; many wonderful phantasies; muttering cries, even to hoarseness and complete loss of voice and speech. Dullness in the head; it is difficult to think; sensation of weakness and lightness in the head; stupefaction of the head with dullness of vision; beclouding of all the senses, after which a red rash (rothe friesels) on the back, with sweating. Staring of the eyes with sleepy aspect; eyes dull and weak; pupil dilated and immovable; with contraction of the pupil, all things appear much smaller and farther removed than they really are. Deafness; illusions of hearing; distortion of the face as if from pain; lips trembling; dryness of the lips. The whole inner mouth as if raw; great dryness of the mouth, so that food taste like straw; dryness of the tongue and mouth, or only of the palate; tongue dry and rough; paralysis of the tongue, or trembling when protruding it; the organs of speech as if paralyzed, with lisping and stammerings; constant mutterings; cries till the speech is lost or he becomes hoarse; complete inability to swallow because of dryness of the throat; sensibility of the abdomen to pressure; blackish diarrhœa every hour; stools smell like carrion; suppressed secretion of urine; retention of urine; copious involuntary discharge of urine.

The above are the symptoms by which these powerful drugs are related to typhoid fever as curatives. In each are symptoms often met in all stages of the disease. Consequently they may be given with confidence whenever their administration is a compliance with the demands of the law of cure. A careless reading of this record may leave the impression that the symptoms of each are little more than a repetition of those of the other. A careful study will show that each has its distinctive character, and it should be well understood that their successful application in practice depends on the recognition and appreciation of this. It is not the plan of this paper to go into a comparison of resemblances and differences of the symptoms of these drugs in detail, though it is earnestly recommended to each student to do this for himself, and to master the subject thoroughly, for by doing this he will have gone far toward making himself master of one of the most dangerous forms of this most dangerous disease. Let him compare the debility, trembling, exhaustion, weariness, and their associated concomitants, of each, so like to these elements of the first stage of the fever, and see wherein each differs from the rest, and he has found the index to its right selection in practice in this stage. So of the symptoms of sleep; intellect; fever, as pulse, sweating, etc.; head; mouth, tongue, and speech; abdomen, stool, urine, and respiration.

We only propose now an allusion to a few of the relationships of these remedies to the fever and to each other, of a general kind, with the hope of facilitating the selection of the right at the bedside of the sick. The first remark we have to make on the class of cases under consideration, is that they present two forms for practical observation and treatment—one of excitement, the other of depression; the first characterized by more or less of violence of delirium, varying in degree from raging madness to quiet visions and mutterings, which just remove cases from the second class, the characteristic of which is *stupidity*. It will be at once seen, if the symptoms of the medicines given above are studied with a little attention, that they vary also in their effects on the organism, as cases of fever do in practice, in the elements of violence and depression; and by a comparison of the symptoms of the remedies given, it will be found that in the manifestation of *violence* they stand in the following order: Bell., Stram., Hyos., Op., Lach.; Bell. being more strongly marked than any other drug; Stram. less, but more than Hyos., while with Op. and Lach. this manifestation is but slight. From Bell. the delirium is loud, talkative, positive; the anger is demonstrative, and strikes, bites, and tears; Stram. is similar, but less in degree; Hyos. is still more mild, while Op., if it rises above the stupid, is still more gentle.

In the second class of cases, marked by *stupidity*, the order in which these remedies stand related is reversed, except as to Lach., which is still in the last place. They stand Op., Hyos., Stram., Bell., Lach. The stupor of Op. is complete. The patient is not to be roused, or only with great difficulty, and to his great annoyance, and even then he comprehends nothing, and immediately falls again into the same unconscious state as before, while at the same time he shows symptoms like the other characteristics of this drug, in the respiration, diarrhœa (watery and offensive), etc. That of Hyos., while it involves loss of consciousness, from which he can be roused with something less of difficulty than from that of Op., does not recognize his relatives or attendants, but soon falls into a gentle though deep sleep, which is likely to be attended with more or less spasmodic symptoms, as jerkings of the limbs, etc., and also with a diarrhœa quite different from that of Op., *i. e.*, yellow, watery, copious, painless, and but slightly if at all offensive. That from Stram. differs from both. The lost consciousness and stupefaction are in large part from the entire occupation of the patient with the visions and images of his delirium, which for the time seem to entirely divert attention from impressions of the senses. The difference between this drug and Bell. is one of degree, in part, the visions of Bell. being more vivid, with less of spasmodic action than Hyos., and the absence of the characteristic diarrhœa, while it may be attended by its own, which is watery, somewhat profuse, and preceded by a copious sweating. The diarrhœa of Stram. is blackish and watery.

Typhoid fever, characterized by predominant abdominal affection, is met by Ars., Carbo veg., Chin., Colch., Merc., Nux mos., Secale, and Sulph. As in the cerebral variety, we propose to group the symptoms of these remedies, and then to ascertain, as far as we may be able, the points which enable us in practice to put each in its place. In the case of Ars., the desire to give the parallel of the group of Griesinger's symptoms with those of the Materia Medica, and also the group by which this drug is related to the fever, entire, will compel a few repetitions. It is hoped the interest of the parallel, and the desirableness of the group entire, will be sufficient to excuse this. The comparison of the symptoms from these two sources is as follows:

SYMPTOMS FROM GRIESINGER.

The febrile symptoms of the patient are aggravated in the evening.

Weakness and prostration are earlier developed and greater than in most other attacks of acute diseases. Very many patients, from the outset, can hardly keep upon their feet.

Dull pain in the forehead, occupit, in the whole head; confusion in the head, vertigo, humming in the ears, intolerance of light.

Sleeplessness, or sleep disturbed by heavy dreams.

Complete loss of appetite, with thirst and bad taste in the mouth, pappy and bitter; the tongue is coated and red at the point and edges.

In the first days there is for the most part constipation, though cases occur where there are fluid stools from the beginning.

Pains in the abdomen are, in this stage, for the most part in the epigastrium.

Pulse frequent, full and soft, and sometimes undulating.

The skin hot and dry; sweating is the exception in this stage.

Urine scanty, dark.

Single or repeated bleedings from the nose.

An increased volume of the spleen is easily detected. Cough and symptoms of bronchial catarrh in many cases.

[The above are Griesinger's symptoms of the first week, in the order in which they occur on this page. He proceeds to say that the fever increases in intensity the second week, and the above symptoms are mostly aggravated.]

The confusion of the head (*eingenomenheit*) is increased till it becomes now a peculiar stupidity.

Speech hesitating and difficult.

Hearing somewhat weakened.

The evening exacerbation brings great restlessness; the night, a moderate delirium.

The mouth and tongue become dry, the latter brown coated.

Rose-red spots, toward the close of the second week, on the chest and abdomen.

ARSENICUM.

Appearance of the symptoms evenings after lying down.

Universal and sudden sinking of the forces. Great weakness, especially in the legs, knees, and feet, on the least attempt at walking, with inability to walk even a few steps without sinking down, or with inability to leave the bed.

Great heaviness in the head, with humming in the ears; stupefying pain in the forehead. Great intolerance of light; vertigo with headache.

Sleeplessness with restlessness and tossing. Many heavy dreams; frightful and anxious dreams.

Complete loss of appetite with severe thirst; bitter, salt, sour, and putrid taste in the mouth; tongue coated white, or red and dry, brown or blackish. (A later stage.) Constipation with pain in the abdomen. Watery and slimy diarrhœa, with great weakness.

Pains of the severest kind in the epigastrium, with great sensibility to pressure.

Pulse quick, frequent, weak, and intermitting.

Heat of skin for the most part dry and burning. Anxious, nocturnal heat, also dry and without thirst.

Urine diminished, with burning.

Copious bleedings from the nose.

Spleen swollen and painful (also in fever). Dryness and burning in the larynx; cough short, dry, deep, fatiguing, with dry excoriation in chest, and expectoration scanty, frothy, and difficult. [A tolerable picture of acute "bronchial catarrh."]

Loss of sensation, consciousness, and speech. He lies senseless, voice stammering and inarticulate. Dullness and weakness of the understanding and senses.

Speech hesitating and slow.

Difficult hearing, as if the ears were stopped.

Night in bed, restless and tossing, with heat, and extravagant, delirious imaginations.

Great dryness and dry sensation of the mouth and also of the tongue. Tongue brown or blackish.

Miliary rash. Red scorbutic spots.

From the above symptoms and their counterpart, it will be seen that Ars. has no second place of importance in the list of the curatives of this fever, especially in its first stage. This will be still more apparent after a careful study of the following:

Anxious weakness, absent-mindedness, staggering gait, with difficulty in walking direct to a given point. General and rapid sinking of the forces. The greatest weakness, especially in the legs, knees, feet, and hands, which tremble, with inability to walk more than a few steps without sinking down. Prostration, with inability to leave the bed; with falling of the lower jaws and eyelids. Emaciation. Petechiæ. Dullness and aversion to all movement; after each disturbance he sleeps again immediately. Sleeplessness with great restlessness and tossing about the bed; sleep is restless and disturbed. Anxious heat and restlessness, with burning, as if hot water were flowing through the veins, or with throbbing in the head, and desire to throw off the covering of the bed. Carphologia; anxious and frightful dreams; cold, sticky perspiration, or sour and offensive. Pulse excited, frequent, empty; or quick, weak, and intermitting; small, weak, and rapid; small and irregular. Pulseless, with excited beating of the heart; anxiety, with tossing in the bed; speaks but little, only complains of anxiety; says nothing, from weakness of body and mind; excess of sensibility to sounds, to talking and light; great indifference to all things, even to life; dull and weak in the head. Delirium. Loss of sensibility—of consciousness—of speech. Delirium, with open eyes; raving, with pain in the head, anxiety, noises before the ears, great restlessness, loss of speech, trembling, and anxious sweating. Stupefying pain in the head, mostly pressing in forehead; great heaviness in the head, mostly in the forehead, with rushing sounds in the ears. Eyes dull, lustreless, prominent, staring, and turned upward; staring, wild expression; contracted pupils. Deafness; ringing in the ears and also in the head. Lips dry and cracked; lips and tongue dry and blackish. Tongue red and dry; cracked and trembling; as if burnt; tastes nothing; great dryness and great sensation of dryness of the tongue, with excessive thirst, though he drinks but little at a time. Thirst for acids, or brandy, or cold water. Great sensibility of the stomach to external pressure; swelling of the spleen, which is painful to pressure; swelling of the abdomen, also excessive. Rolling and gurgling in the abdomen, as if from much flatulence. Putrid, offensive flatus; involuntary and unnoticed stool. Diarrhœa with colic pains; with great weakness; yellow, watery, and small; greenish, dark brown, with the offensiveness of foul ulcers; putrid; black, burning, excoriating stools, with restlessness and colic. Involuntary urination; diminished urine, which is burning; very turbid; greenish, dark brown, turbid when passed, and does not become clear on standing. Oppressed respiration with extreme prostration.

*Carbo vegetabilis.*—Weakness, especially of the legs, in the evening; or in the morning, with sluggishness, and disposition to perspiration and trembling of the limbs. Trembling of the body, with prostration. Debility as if after a severe sickness; the joints seem too weak to carry the body; pulse weak; entirely pulseless; disposition to great perspiration. The mind is sluggish, with inability to think; ideas move slowly and constantly around one object, with confusion of the head (*eingenommenheit*), as if bound fast. Vertigo the whole day; whirling, while moving, especially the head; heaviness of the head, like lead; in the forehead, with dull pain. Heaviness and immobility of the tongue, with difficult speech; heat and dryness of the tip of the tongue; great sensibility of the region of the stomach to pressure; distention of the abdomen, as if from flatulence, especially in the afternoon; constant distention with copious escape of flatus; rumbling and gurgling in the abdomen; fermentation in the bowels, with subsequent stool like diarrhœa and discharge of putrid flatus; involuntary stools which are offensive like carrion; urine turbid and reddish; dark colored; strong smelling.

*China.*—Sense of internal illness, as of impending disease; painful weariness in the limbs, as if after a long walk, or exhaustion from loss of fluids, with constant inclination to stretch them or change their position. Heaviness in all the limbs, especially of the thighs; aversion to all efforts of body or mind; weakness, with relaxation of body and mind; with insensibility; he can hardly hold the head erect, and falls asleep; weariness; aversion to movement. Great weakness with strong disposition to perspiration during movements and in sleep; great sinking of the forces; unconquerable disposition to sleep, with weakness; frightful phantasies, in the evening, in bed, with frightened starts on closing the eyes to sleep; anxiety on waking from frightful dreams; insensibility on waking, or vertigo, which is increased by raising the head; indifference and apathy; taciturnity; obstinate silence, will answer nothing; angry, quarrelsome disposition; nervous irritability, with depression of spirits, and intolerance of all impressions on the senses, especially of noises; slow movement of ideas, and also of the power of comprehension. Vertigo, with nausea and subsequent heat; heaviness of the head, with increasing vertigo, mornings, or waking, with weakness of the limbs; pupils much contracted or dilated and insensible to light; countenance pale and sunken; hippocratic; with sharp nose, and hollow eyes with dark circles around them, with insensibility and indifference. Lips dry, hard, and cracked; blackish lips; blackish tongue; cracked; as if raw or burnt. Swelling and hardness of the spleen; pains in the abdomen as if there would be diarrhœa; distention of the abdomen, with pains and diarrhœa; with hardness and pain; with constant tension; in the morning, tympanitis. Rumbling in the bowels, especially in the upper part of the abdomen; thin stools like diarrhœa; yellow, watery; slimy, involuntary, thin, and yellow; blackish; with the respiration rattling, and moaning sounds in the chest, and loud sounds through the nose.

*Colchicum.*—Great weakness and sensibility of the surface; weakness, as if after exertion. If the patient be raised up the head falls constantly backward and the mouth opens to the widest extent. Sudden sinking of the forces, so that in ten hours he can hardly speak or walk; cadaverous aspect and extreme prostration; emaciation; lying on the back, comatose; eyes half open; respiration audible and accelerated; hands and feet cold; trunk hot and extremities cold; skin dry; sweating; suppressed cutaneous transpiration; forehead covered with cold sweat. Pulse small and contracted; quick and hardly perceptible; small and frequent; quick and thready; pulseless. Delirium with cephalalgia; intellect beclouded though he gives correct answers to questions; unless questioned he says nothing of his condition, which does not seem to him dangerous. Perceptions entirely lost; he is unconscious; carphologia; eyes hollow, staring, and sunken; pupils much dilated and little sensitive to light; immovable and but slightly dilated; left pupil contracted while the right is dilated. Nostrils dry and black. Face sunken and hippocratic; risus sardonicus; lips, teeth, and tongue covered with a thick brown coating; lips cracked; face covered with perspiration; grinding of the teeth; tongue protruded with difficulty; tongue bright red; tongue heavy, stiff, and numb; loss of speech; inextinguishable thirst; epigastrium and stomach extremely sensitive to pressure; abdomen distended, tense, and hard; surface of the abdomen hotter than the rest of the body; tympanites with pain in the back. Watery diarrhœa, the stools are passed insensibly; stools fluid, offensive, with white flakes; involuntary stools; numerous, liquid, black, offensive stools, with severe pain; secretion of urine suppressed; urine copious; involuntary urination; respiration irregular and intermittent.

*Mercurius.*—Great weariness in all the limbs; weakness, in the evening, with depression of spirits; great weakness on the slightest movement; in the morning, with nauseating sinking, heaviness of the legs and drowsiness; intolerable weakness, with giving way of the knees; with sensation as of lead in the veins, worse when sitting; attacks as if body and mind were unstrung (entschlaffung). Aversion to speaking, with confusion (wuestheit) of the head, so that he can neither read nor perform the least labor, but falls asleep while sitting; weakness and exhaustion, with heat, rush of blood, and trembling, from the least labor; much exhausted after a stool, with griping in the abdomen; weakness, so that he is ready to fall, with inexpressible sense of illness in body and mind, which compels him to lie down. General exhaustion of forces, with emaciation; small, transparent vesicles filled with watery fluid, appearing in the morning on various parts of the body (Sudamina). Too great disposition to sleep, which is too profound; he slept twelve hours and would longer if not waked; with extreme prostration and sleepiness, he is unable to sleep; frequent waking, as if from fright or from wakefulness, with much tossing; intolerable restlessness, anxiety, and discomfort at night in bed, with sleeplessness; on waking, whirling in the head, by which sleep is made more distressing than pleasant; frightful dreams, as if he fell from a height, or as if he were not in his own home, and talking of distant villages; of shootings; of street robbers; frightful imaginations prevent his sleeping, in the evening. Great perspiration at night, also it is greasy, stiffens the linen, stains it yellow, or is offensive. Great indifference, so that he takes no notice of anything; has no desire to live, wishes rather to die, with indifference to the most loved objects. Loss of thought for the moment; insensibility, so that he knows not where he is; loss of consciousness and speech, with pulselessness, with cadaverous aspect, while the natural heat of the body is retained, and he is in a sleep-like state, from which he emerges with consciousness and speech. Vertigo while lying on the back, like whirling and weakness (weichlich), better when lying on the side. As if the head were bound with a band, with pressure. Pressing headache in the occiput, or outward pressure in the forehead, with pain in the bone over the eyebrows, especially when touched. Dullness of the eyes; pupils dilated; deafness. The ears as if stopped, with rushing sounds. Bleeding from the nose, in sleep. Pains in the corners of the mouth as if cut; excoriation and cracks in the corners of the mouth; ulceration of the corners of the mouth, with pains as of excoriation. Spongy gums, separating and easy bleeding; bleeding of the gums on the slightest touch. Tongue very rough; brown or blackish; painful, as if chapped and burning; dry and hard. Great thirst day and night for cold drinks, especially for water. Great sensibility of the stomach and epigastrium to touch; fullness of the epigastrium with tension, and embarrassed respiration. Distention of the abdomen, with hardness or with painful sensibility to touch; rumbling in the bowels after every drinking, or before every stool; stools dark green and frothy; brownish, soft, floating on the water; pitch like, sticky; urine offensive, first clear, then white as if mixed with chalk.

*Nux moschata.*—General restlessness in the muscles, with vertigo; anxiety in the body with disposition to trembling. Pain in the neck, bones, and generally as if after taking cold in copious perspiration, with pressing to the forehead. Loins and legs as if bruised and weak; after the slightest exertion weakness with inclination to lie down. Bluish spots on the skin. Great sleepiness with giddiness, as if drunk, so that she does not know where she is, and walks with the eyes shut. The profoundest coma, lying silent, immovable, insensible; a dreamy state, with drowsiness, and falling of the eyelids; restless sleep at night. Absent-minded and insensible, as if intoxicated; insensibility and giddy vanishing of thought; slow movement of ideas, he dwells long on his answer before giving it, and often he answers not at all. Delirium and stupidity; frantic drunkenness; drunkenness, also with staggering, or indolence, or heaviness in the head, and pain first in the forehead then in the occiput. Dryness of the mouth, tongue, and throat, with fullness of the stomach and loss of appetite; in the evening, so great that the tongue sticks to the roof of the mouth, yet without thirst. Distention of the abdomen as if from flatulence; tension of the abdomen, with restless sleep, or heaviness in the upper part of the abdomen. Rumbling, rolling, and gurgling in the bowels; discharge of fetid flatus; watery stool instead of flatus, after a previous constipation and hard stool; colliquative, putrid diarrhœa. Urine scanty, very high colored, and clear.

*Secale corn.*—Great general weakness, or sometimes more in the upper and sometimes in the lower extremities. Heaviness of the limbs with numbness; weakness of the limbs; rapid sinking of the forces; fainting; great trembling of the part moved in every effort, even of the protruded tongue. Rapid emaciation; petechiæ; great inclination to sleep, like coma; deep sleep and long continued; stupefying slumber the whole day; sleep at night is restless with heavy dreams; after sleep is much exhausted. Pulse small and contracted (zusammengezogen), slow, small, and intermitting. Sweating from the head to the epigastrium; profuse and general; cold and also sticky. Depression of spirits constant, with timidity; great indifference to everything. Delirium bland or violent, like mental aberration, followed by vomiting and this by deep sleep; after the delirium still greater vertigo, like intoxication, with sense of lassitude and weakness; the boy understands nothing, and answers no questions; continued stupidity with dilated pupils; difficulty of thought and speech; diminished sight and hearing, or entire loss of these senses; confusion (wuestheit) and stupefaction of the mind; drunken vertigo, staggering and inability to stand erect. Distortion of the eyes, with pupils nearly closed; rolling of the eyes; eyes wild and wandering; staring of the eyes; squinting; pupils either much contracted or dilated. Deafness, with humming and rushing sounds in the ears. Bleeding from the nose. Face sunken, hippocratic, pale, and expression dull. Dryness of the mouth, with thirst; tongue discolored, brown, or comparatively black; speech difficult, weak, indistinct, or stammering; slow and weak, as if the organs had an impediment to overcome. Dryness of the throat. Painful sensibility of the stomach and epigastrium; abdomen distended, tense, hard, and painful if pressed; painful diarrhœa, with great sinking of the forces; putrid, extremely offensive, colliquative diarrhœa; involuntary diarrhœa. Suppression of the urinary secretion; difficult urination, with constant desire. Voice hoarse and hollow; anxious and difficult respiration, also with sighing and hiccough; loss of speech and subsultus.

*Sulphur.*—Very heavy and weak in the limbs from morning to evening; lassitude the whole day; vanishing of the power of the arms and legs, like fainting; he was near losing his consciousness. Always fatigued and weak; weary, as if after sickness; much prostrated, weak, and averse to effort, even to speaking; weakness of the limbs, with trembling after every effort. Very weak and sleepy the whole day; unconquerable sleepiness in the day-time; even when seated at work he cannot prevent sleep. Restless tossing at night in bed; restless at night, he wakes from each sleep with fright, as if from a terrifying dream, and after waking anxious phantasies, as from ghosts, or his employments, from which he cannot at once free himself. Restless sleep; full of dreams; before midnight, irrational talk in his sleep like delirious dreams. That which he has dreamed seems to him a reality. Snoring in his sleep; sleep with half-open eyes; unintelligible mutterings in sleep; frightful and anxious dreams every night. She has no rest day or night. Great absence of mind; he cannot fix his attention on present objects or manage his affairs. Indolence of body and mind, and through the day is averse to employment or movement; in the evening he is averse to work, pleasure, speech, and movement; he is extremely uncomfortable and knows not what is the matter with him; he is dull in his senses, stupid and embarrassed, and avoids moving about. When spoken to, he is as if wakened from a dream, he appears silly, and can only comprehend and answer after great effort; he cannot bring two ideas into connection, and is as if weak in his intellect; great dullness and vacuity; sensation of fullness and heaviness in the head; heaviness in the vertex; pupils contracted; increased sensibility of hearing; every sound annoys him. Dryness of both ears, which rapidly disappears; bleeding from the nose for seven days. He can endure no odors; pale, sickly aspect, as if after long illness, with great uneasiness; sunken eyes with blue circles around them; dryness of the lips; cracked lips; trembling of the lips; brown mucus is deposited on the teeth; bleeding from the teeth and gums. Offensive smell from the mouth morning and evening; tongue very dry in the morning. The stomach is very sensitive to touch, even the bed covering causes pain. The region of the liver is sensitive to touch; distention of the abdomen; swelling and hardness of the abdomen in the evening; tension of the abdomen, as if from pent-up flatus; rumbling, rolling, gurgling, grumbling in the bowels; much flatus; offensive flatus; diarrhœa like water every half hour, each after grumbling in the bowels, without pain. He has six discharges like diarrhœa, then he faints; first he had heat and warm sweat, then cold on the face and feet, with white tongue; frequent and frothy diarrhœa, with tenesmus, even at night. After the stool great prostration; urine scanty; dark brown urine; the urine becomes turbid after standing an hour; very offensive urine.

These are the principal symptoms by which the above medicines are related to typhoid fever. As to Nux mos. and Secale, the brain symptoms are so considerable as to make it perhaps a little doubtful whether they would not be more properly considered in connection with the mixed group. They have been presented here because of the importance of the *character* of their abdominal symptoms, rather than of their great number, and because the fevers to which they are appropriate are dangerous chiefly through the progress of these symptoms.

Ars. is second in importance to no drug in the treatment of this fever. This is true if we have reference to the proportion of cases calling for its administration, or the grave character of those in which it is often found curative. It is not, however, to be given without due discrimination, if we would avoid evil from its use. In the first place, from the symptoms above given, it is quite apparent the cases requiring Ars. are characterized by *anxiety* and not by *coma.* Though the patient may "fall asleep again immediately after being roused," his sleep is not comatose.

*Restlessness and nervous erethism* are also marked features of these symptoms of Ars., and consequently of the fevers to which it is applicable, especially in the first stage. In these elements it stands alone among the group of drugs related to the typhoid state. It is rare that Ars. is appropriate to the treatment of any acute disease when the prevailing disposition is to a quiet repose, and never in the early stage of this fever. In a later stage, the patient may be *quiet,* and yet be a proper subject for Ars., but the quiet is that of exhausted vital force, which is rather tending to dissolution than healthy repose. He may in this stage be insensible, but this is from exhausted brain power, coincident with the loss of power and function in all the other organs of the body, and not from congestive coma. And even here, if roused, there is so much of the characteristic anxiety and restlessness as the patient has power to show. In relation to the *diarrhœa* of the cases which call for Ars., it may be remarked that this element is likely to be developed early in the history of the case, and each discharge to be followed by marked increase of the exhaustion. The discharges are decidedly offensive. The swollen abdomen has not the tense, tympanitic character of that produced by China. It is softer, though full, and gives out more frequent and marked sounds of moving flatus and liquid in the intestines. The flatus with China is pent up, and the noises from its transit are less. In a late stage of the fever, if sinking, threatening dissolution, occurs, Ars. is one of our chief reliances. In cases showing the characteristic anxiety and restlessness, with cold, sticky perspiration ; rapid, small, and weak pulse ; or large, soft, and slow, Ars. will often succeed in arresting the downward progress of the case and in raising it to convalescence. It is not to be used in cases of sinking indiscriminately. It is not often indicated in cases where body and mind are quiet. When the exhaustion has been preceded by hemorrhage, or great loss of fluids, as by serious diarrhœa, China will be preferable, but then the characteristic restlessness and anxiety of Ars. will be wanting. If, in the absence of these, and after protracted illness, the sinking be sudden and characterized by coldness or cold perspiration over the whole body, or only on the trunk, with slow, small, and soft pulse, Verat. is indicated. If the exhaustion be very great, with coma and loose rattling respiration, cold respiration on the extremities and face, pulse small and weak, or the patient is even pulseless, Carbo veg. is better. These cases require the most careful individualization, and comparison of their symptoms with those of the drug, before selecting the remedy, for there is not likely to be time for the correction of a mistake if this be made at the first effort. Where the exhaustion is from progressing decomposition of the blood, this process is more likely to be arrested by Lachesis.

*Colchicum*, if rightly applied, will be found a remedy of considerable value. A cursory glance at the symptoms given above is all that is required to see its true position in the group in which we have placed it. Its almost entire want of brain symptoms places it next to Ars., if it be not even less characterized by these than that remedy ; while in its abdominal symptoms it much resembles China. It may be regarded as occupying ground between these two. If we meet with the tympanitic distention of the abdomen so characteristic of China, with watery diarrhœa and exhausted forces with even a greater freedom of intellect than with Ars., with the absence of the anxiety and restlessness so characteristic of this last, we have a case that just calls for Colch. and for no other drug ; and if not given too freely, it will not disappoint our hopes of success.

The symptoms of Sulphur here given are worthy of study. In the general symptoms there is little disclosed, except debility, to connect the drug with the typhoid state. This is certainly very like what is often met in the *early* stage of this fever. So of the sleep and dreams. The mental symptoms are rather such as are likely to result from a certain degree of general loss of vital forces, in which the brain as a part of a whole participates, than such as indicate any special invasion of this organ. With this the head symptoms are in full accordance. The organs of sense appear in a state of erethism from participation in the same general condition. But a cursory glance at the symptoms of the alimentary tract and its associate organs is sufficient to disclose that here is a strong resemblance to the phenomena developed by the grave forms of the fever in the same sphere. Those of the lips, mouth, teeth, stomach, abdomen, and stool are quite worthy of notice.

But *Sulphur* has a wider range of relationship to the treatment of this fever than that of simple resemblance to its symptoms found in the above record. This exists in its antagonism to chronic miasms called by Hahnemann, in one word, *psora* (and which the reader may call by a better name if he has one), so likely to become active during the invasion of any severe attack of acute disease, and in this fever more than in any other, increasing greatly the difficulty of the cure, and often rendering this impossible, till after the use of remedies specifically antagonistic to these miasms. Among the remedies so related, Sulph. stands pre-eminent. In such cases as resist the action of the drug whose symptoms on the healthy are most like those of the fever, after a proper trial (and many such cases there are), there need be no hesitation in giving a single dose of this remedy, which will often be followed by a remarkable change in the state of the patient for the better. This use of the *anti-psoric*, to meet the demand of the complex nature of the case, is not to be confounded with nor raised as an excuse for that "alternation of remedies" against which we have protested in this paper. In this permitted and beneficial use of an *anti-psoric*, supplementary to the leading curative of the case, and that "alternation," there is nothing in common except both involve the use of two drugs. These two courses are entirely different both in principle and result. This complex condition of the fever has also often been successfully met by a remedy but little known to many practitioners, it is believed, but which nevertheless has great value in just this class of cases. The reference is to Psorinum.

In illustration of the action of these two remedies, the following case, which occurred in the practice of the writer some years since, may serve. The patient was a girl, ten years of age, light complexion, slender form, temperament mixed nervous and lymphatic, the mother of whom had suffered from often repeated attacks of facial erysipelas, while the father would be at once classed with the scrofulous by those who use the term. There was in the early stage no notable characteristic, except the great rapidity with which the case reached the state usually met in the last stage of the severer forms of the fever. On the sixth day, through an uninterrupted downward course, the patient had come to utter insensibility; constant profound coma or lying with staring eyes; involuntary and unnoticed evacuations into the bed of both urine and feces; subsultus; when the eyes were open, reaching after objects in the air and picking at the bedclothes; entire loss of hearing, and apparently of sight; intestinal evacuations, liquid, brownish, and extremely offensive; pulse small, weak, quick, and 130 per minute. In this alarming state of things, in accordance with the advice of my friend, Dr. A. F. Haynel, the patient got four globules of Psorin.[30]. In twelve hours, having had no other dose and no other medicine, she answered questions loudly put, the diarrhœa was less frequent. Pulse 120 per minute. The dose was permitted to act and the improvement to progress till the end of forty-eight hours, when she fell again into insensibility and involuntary evacuations, with an increase of the other remaining symptoms, though they were slighter than before the dose of Psor. was taken. She now got Sulphur, third trituration, half grain. The amendment which followed was prompt, the convalescence rapid and complete. The patient required and got no other dose and no other medicine. There can hardly be a doubt that in this case, if there had been a continued reliance on and use of what seemed to be the appropriate remedy in this case, regardless of the psoric complication, it would have speedily reached a fatal termination. Whether the cure is to be referred to the action of these two drugs solely, or whether by extinguishing the psoric element the case became amenable to, and came under the power of, the remedies previously employed is a question on which opinions may differ. The recovery was rapid, complete, and unexpected.

The third variety of typhoid fever, that in which neither the cerebral nor abdominal symptoms can be said to preponderate much, and of which we have presented Bryonia as the drug representative, may require Arn., Bry., Calc., Nux vomica, Pulsatilla, Rhus tox., or Verat. The following are symptoms of these drugs, the likeness of which may be met in our practical dealing with this class of cases:

*Arnica.*—Great heaviness of the limbs, as if from extreme fatigue; weakness, weariness, and bruised soreness, which compels to lie down; lassitude and sluggishness in the whole body; general sinking of the forces. Sleep unrefreshing and full of dreams. In sleep, whimpering, loud talking, loud blowing (schniebendes) in and expirations; involuntary evacuations of fœces and urine; anxious and heavy dreams from the evening into the night, which much affect the body; frightful dreams. Sits as if in thought, yet thinks of nothing, like a waking dream; forgets the word while speaking; loss of consciousness; delirium; stupefying confusion of the head, like a heaviness in the forehead; vertigo while raising or moving the head. Pupils contracted, with beclouding of the head. Bleeding from the nose. Lips dry, as if parched by thirst. Dryness of the mouth, with great thirst. Putrid smell from the mouth. Distention and hardness of the abdomen. Brown or white diarrhœa; diarrhœa at night, with pressure in the abdomen as if from gas; with distention of the abdomen before the stool; with rumbling in the abdomen during the stool.

*Bryonia.*—Great and general weakness on waking from sleep; with sluggishness, lassitude, and drowsiness; he feels better when lying down. Loss of strength on the least exertion, especially after rising from sitting and at the beginning of walking, and with want of firmness in all the joints. Weariness and heaviness in all the limbs, especially the legs, and on rising from sitting. Great sleepiness through the whole day; he must sleep all the time; comatose sleep with anxious delirium, or with dry heat, jerkings of the face, and involuntary stools. At night, in bed, he lies without consciousness, with groans, cold sweat on the forehead, followed by weakness; thirst, with frequent drinking; delirium on waking from sleep. In sleep, whimpering, at three o'clock A. M; distortion of the mouth or movements like chewing; involuntary stools; anxious dreams, with waking with fright and outcries; frightful, delirious dreams, as if cut and hacked by soldiers, with desire to escape. Wakes weary and unrefreshed; anxious perspiration, which prevents sleep, with sighing, short cough, and pressure on the chest. Acid sweating at night, preceded by thirst, with pressing drawing in the head toward the end of the sweating, followed by confusion of the head. Inclination to escape from the bed; disposed to be timid and fearful; dejected, debilitated, with aversion to thought; irritable, peevish, and easily offended. Debility of the mind, with vanishing of the thought, like fainting; dullness of the head, with difficulty of thinking and great forgetfulness; delirium, especially at night; or in the morning, upon business affairs, with disposition to run away; in the evening, with hasty speech, imagining he is under control of strangers, and desires to go home. Stupefaction of the head; feeling of drunkenness, with desire to lie down, or with rush of blood to the head; great heaviness of the head, also with pressure in the brain, and desire to lie down; fullness of the head with vertigo; throbbing in the head, in the forehead and occiput, worse when moving. The pains in the head are increased by moving or opening the eyes. Bleeding of the nose, especially at three o'clock A. M., or after rising; daily, for many days; in sleep. Face pale; yellow; heat of the face, especially in the evening, also with burning and redness, especially of the cheeks. Dryness of the mouth with great thirst, or also without thirst. Tongue dry, rough, or dark colored. Indistinct speech from dryness in the throat. Violent thirst day and night, does not drink often, but much at a time; thirst for cold drinks; for wine, for coffee, or acids. The least pressure on the stomach is insupportable; excoriating pain in the epigastrium from touch and cough. Pain in the abdomen, as if there would be diarrhœa; distention of the abdomen; rumbling sounds in the abdomen, especially in the evening in bed or at night. Diarrhœa every three hours with sudden and almost involuntary discharge; with weakness, which compels to lie down, especially in the morning or at night, with burning in the anus; offensive, like putrid cheese; pain in the abdomen before the diarrhœa. Dry, short, hacking cough, evening in bed, as if from roughness or dryness in the larynx.

*Cocculus.*—Attacks of jerkings of the muscles, especially of the lower limbs. Trembling of all the limbs. He can hardly stand erect on account of great weakness; great weariness at nine o'clock A. M., with heaviness in all the limbs and almost an unconquerable inclination to sleep. Indolence (trægheit), and he sits in silence. Wants to lie down. Faintings, also especially from bodily movement, with spasmodic distortion of the facial muscles. The greatest weakness. After the slightest exertion he must sit down. The least effort, or interruption of sleep, is followed by great loss of strength. Unconquerable coma vigil. Coma. The greatest irritability; can endure neither noise nor contradiction. Dullness in the head, also with cold sweat on the forehead and hands. Vertigo, like drunkenness, with dullness in the forehead, as if a board were before the head; whirling vertigo when rising up in bed, with nausea, compelling to lie down again. Pupils contracted or greatly dilated. Dryness of the mouth at night. Tongue rough, and also dry, with whitish-yellow coating. Great distention of the abdomen. Rumbling in the intestines. Frequent urination in small quantities.

*Nux vomica.*—Great weariness also immediately upon the slightest movement. Great inclination to lie down or to sit. Sudden sinking of the forces. Sudden, paralytic loss of strength, even in sitting, but most when moving. Immoderate sensibility to the open air. Uncommon sleepiness, as if from stupefaction of the head. The night seems long and tedious, with comatose slumbering and dreams full of bustle and hurry. In sleep: wakes in fright from the least noise; moaning and whimpering; blowing (schniebendes), whistling expiration through the nose; snoring inspiration, as if the posterior nares were contracted, before midnight; delirious phantasies on lying down; half waking, sad phantasies of the headless bodies of dead acquaintances; in an evening slumber he springs delirious out of bed; dreams full of frightful images; delirious and extravagant dreams; wakes as weary as when he slept. Chill on the slightest movement. Restlessness, with dilated pupils. Increased sensibility to all impressions. Sounds, talking, odors, and light are insupportable. Silence as if averse to everything. Stupefaction of the head; drunkenness and beclouding of the head; vertigo, with obscured vision and ringing in the ears; vertigo as if in the brain there were a whirling in a circle, with momentary loss of consciousness; vertigo on rising from lying on the back, with obscured vision. Heaviness in the head in the morning with drunken vertigo. Bruised pain in the head, as if compressed. Throbbing in the vertex on endeavoring to fix the attention on an object. Contraction of the pupils; dilated pupils, with slow respiration. Continued bleeding from the nose. Dryness of the nose. Offensive smell from the mouth, putrid, like carrion; dryness of the mouth also, only of the forepart, and especially of the tip of the tongue, or in the morning, as if from the use of spirituous drinks the evening before. Tongue black and cracked, with deep red edges; brownish tongue. Inability to speak loud; while speaking, sensation as if the tongue were too thick. Food is without taste. Thirst, especially in the evening, with disgust for water. Great sensibility of the stomach and epigastrium to pressure. Distention of the epigastrium, with painful sensitiveness to touch. Pain in the abdomen as if all were raw; distention of the abdomen immediately after drinking. Frequent, small, diarrhœa like stools, which are excoriating to the external parts; diarrhœa putrid, watery, with cutting and drawing pains in the abdomen and loins, extending to the thighs.

*Phosphorus.*—Sluggishness of the limbs, more in the forenoon, with heaviness; heaviness and dullness of body and mind; loathsome (widriges) sensation of the whole body, with weakness of the joints, especially of the knees, while sitting and in quick motion. Sense of illness and discomfort in the whole body, especially in the stomach, even in the open air; general relaxation, with great nervous weakness; weariness in the whole body, especially in thighs; frequent sudden attacks of great weakness; walking affects him much, even the least walk produces great fatigue and headache; loss of all strength; trembling in the morning with jerking of the limbs. Great sleepiness, like coma; stupefied, as if drunk; dizzy, staggering, on waking from sleep; frequent waking from sensation of heat; dry heat with pain in the part on which he has lain, as if the place had been too hard; great heat with dryness of the mouth, which impels him to drink; constant dreaming, with great restlessness at night; dreams of animals biting, with outcries, and waking with anxiety. Pulse accelerated, full, weak, and small; throbbing in the arteries of the throat. Increased sensibility of the senses, especially of hearing and smell. Indifference to everything, even the most loved child. Slow movement of ideas; absence of ideas; delirious phantasies in slumber and waking, as if she were on a distant island, had great occupation, was a lady of rank, etc.; as if stupid and disconcerted (verduezt) for many days; he cannot comprehend any idea, with headache; painful stupidity on waking in the morning; vertigo, with confusion (eingenomenheit) and stupidity of the head as if he would lose his senses. Throbbing in the top, left side, or back of the head; in the temples often for a half hour; humming and buzzing in the head almost the whole day. Frequent and copious bleedings from the nose; copious bleeding in the evening. Sick, pale aspect, especially in the evening; hollow, sunken eyes, with blue circles, and pale, or earth-colored, sunken countenance, dingy colored, hippocratic face. Dry lips, with dryness of the palate. Bleeding of the gums from the slightest touch. Intolerable dryness of the mouth, sticky, with great thirst, which is not relieved by drinking. Tongue as if coated with a skin; as if burnt and rough at the tip; dryness of the tongue. Dryness of the throat, which hardly permits swallowing. Constant thirst for water. Pain as from excoriation, or as if inflamed in the hypogastrium, especially when touched, with weakness; sensibility of the abdomen below the navel when pressed on; distention of the abdomen, with pain as if bruised, in this part and in the loins, when touched; extreme sensibility of the abdomen; tension of the abdomen from accumulated gas, though much is discharged; hardness of the abdomen, with much flatulence; very full, tense, and hard abdomen. Rumbling in the bowels, also painful, as if there would be diarrhœa; very offensive flatus. Diarrhœa—black, gray, involuntary. Urine of strong ammoniacal odor, turbid, which deposits a white sediment; sharp, disgusting smelling urine, like the smell of violet roots. Dry cough, with pain in the head as if it would burst; severe cough, with pressing headache the whole day; troublesome cough which causes pain in the forepart of the chest and wakes from sleep; loose cough without expectoration, with pain and sense of excoriation in the chest, so that he fears to cough. Shortness of the breath, also with vertigo, or with great anxiety (beklemmung), or also after each cough; anguish in the chest, as if pressed together, with want of breath; pressure on the lower part of the chest.

*Pulsatilla.*—Sluggishness (trægheit), with constant desire to sit or lie down. Heaviness of the whole body, sometimes great especially of the arms and legs, with chilliness. Inordinate weariness from a short walk. Weariness of the legs even while rising from long-continued sitting. Weakness of the whole body, which compels lying down; can walk but a few minutes on account of weakness, and is often through the day compelled to sleep whole hours. Trembling weakness. The weakness and weariness of Puls. develop themselves mostly as heaviness. Light sleep, with the feeling on waking that he has not slept at all; restless, stupefying, dull sleep, with constant tossing about; slumbering long continued, full of phantasies and dreams; great restlessness and tossing in the bed, as if from great heat, or throwing off of the bed covering because of heat, with heat of the palms of the hands; he casts off the clothes because they are too tight or too warm, yet he shivers as soon as he is uncovered. External warmth is unsupportable; warm sensation, as if in an overheated room, or as if hot air were blowing on one, which excites headache. Heat of one hand with coldness of the other; heat of the hand and foot of one side, with coldness and redness of the other, evening and night. Pulse quick and small; weak and almost suppressed. Great disposition to perspiration during the day; on the right side of the face; on one side of the body, either the right or the left, cold perspiration with trembling of the whole body. He falters and hesitates in his speech, and only answers with indignation. Unconsciousness; he knows neither where he is nor what he does; dullness, like a want of memory; great difficulty in speaking, to use right expressions; fixed ideas; when he has once grasped a thought, it cleaves to him and will not vanish; great crowd of changing ideas; nocturnal delirium; violent delirium with loss of consciousness; terrific visions, with fear and desire to hide or run away; dullness of the head with heaviness; confusion of the head with vertigo when moving; dullness of the head, with bruised pain in the forehead; vertigo, like drunkenness; vertigo so that he cannot rightly comprehend an idea; heaviness of the head, so that he has difficulty in raising it; heaviness of the head with intolerance of light. Pain in the head as if the brain were torn, in the morning, on waking, and after. Pupils first contracted then dilated. Deafness, as if the ears were stopped; with rushing sound, like the wind. Cracked lips. Putrid smell from the mouth, morning and night; foul smelling slime covers the mouth in the morning on waking, with dryness of the mouth and throat; tongue as if burnt and insensible; cracked, with gray coating. Throbbing in epigastrium and stomach. Rumbling (knurren) in the bowels, also with diarrhœa stools and pinching and grasping pains in bowels. Watery diarrhœa, especially at night; unconscious stools at night, in sleep. Red urine; dark red, without sediment; brown, also with burning; brownish red.

*Rhus toxicodendron.*—Great weakness in the whole body; with sensation as if bruised, lasting the whole night; with constant desire to sit or lie down, with soreness in all the bones; will lie down, sitting does not suffice; on sitting up he has nausea. Petechiæ, with great weakness, even to the loss of all strength; lenticular red spots with small vesicles in the centre. Sleepiness the whole day, with anxiety, restlessness, sadness, dry lips, and a constant desire to lie down; sudden sleepiness in the evening, so that she cannot rouse herself from it; paralytic sensation (lahmigheit) in all the limbs; constant comatose slumbering (schlummersucht), full of troubled and intermittent dreaming; snoring, murmuring, and picking at the bedclothes; restless, disturbed sleep, with frequent turning and throwing off the bed covering; great restlessness at night in bed; great anxiety and fearfulness, which drives one from the bed; he must spring out and call for help, on account of indescribable feeling of distress; in sleep, open mouth; very short breath. Violent delirium, with severe pains in the limbs, great weakness, dry tongue (red or black); dry, brown, or black lips; heat and redness of the cheeks, carphologia, pulse quick and small, lethargic slumbering, with murmurs and snoring. [The above group is clinical, not pathogenetic ] Sweating over the whole body, or only on the face, which is hot; in bed, in the morning, over the whole body except the head; gentle sweating, during which he wishes to be covered. Great anxiety, with pressure in the heart and tearing pains in the loins; with sinking of all the forces, more after than before midnight. Absent-minded, as if absorbed in thought, and yet a want of ideas; illusions of the phantasy and visions; delirium, also loquacious; prostration of the mind, he cannot bring two thoughts together, as if quite stupid; thought is difficult, and he is averse to speaking; slow movement of ideas; cannot remember the most recent occurrence; weakness in the head; if he turns he loses his consciousness for the moment, and after stooping he cannot rise; while sitting, stupid, as if drunk, on rising, dizzy, as if he would fall forward or backward; head is stupid and dull; confusion of the head; stupid weakness of the head; vertigo, as if drunk, and as if he would fall, after rising from the bed. Headache when opening the eyes, when waking from sleep, as if the brain were torn, as if after a brandy debauch, worse when moving the eyes, then in the occiput, as if the cerebellum were bruised, and outward pressure in the temples; as if the two sides of the head were pressed together. Great sensibility of the scalp to touch, like a boil. Bleeding of the nose at night, in the morning, sense of dryness or actual dryness in the mouth, with severe thirst not relieved by drinking, afternoons and after midnight. Tongue not coated but very dry, with desire for drink; dry and hard, red or brown tongue. Repugnance to all ingesta; he tastes neither food nor drink. Distention of the abdomen, in the region of the navel, with severe pinchings; painful, with pain as if from pent up gas; very offensive flatus. Diarrhœa, sudden, thin, yellow, frothy, almost without fetor or preceding colic, but the discharge is involuntary, as if from paralysis of the sphincter; nocturnal, with severe colic, which disappears after the discharge, or with headache and pain in all the limbs; involuntary stools, especially at night in sleep.

*Veratrum.*—Weakness in all the limbs; inclination to lie down; weariness, as if after long walking; weakness as if from overheated air; he sinks down exhausted; universal weakness in the morning, as if after too little sleep; sudden sinking of the forces with disposition to sleep; he cannot stand up but only lie or sit; if he stands he has the greatest anxiety, with nausea and cold sweat on the forehead. He sleeps on his chair, in a half conscious state; stupefying sleep, like comatose vigil (wache schlummersucht), with frequent starts as if from fright, and one eye open and the other closed or half open; uninterrupted sleep for three days; sleep too profound; frightful dreams, with subsequent vomiting of green, tenacious slime; angry dreams; anxious; as if bitten by a dog and he cannot escape; as if he were hunted; of robbers, with frightened waking, and a fixed idea that the dream is true. General heat with sweating, especially in the evening in bed, or during the day, with pale face. Great thirst for cold drinks; afternoon and evening, cold sweat on the whole body, or only on the head and trunk, or only on the forehead; cold and sticky; nocturnal, also continued, and especially in a long sleep. Great indifference, with a kind of insensibility which impels to rubbing the forehead. Silence. He is averse to speaking, and his voice is light and weak; he permits no one to speak to him. He remembers events only as dreams; almost entire loss of mind; delirium, also violent; mild, with entire coldness of the body, open eyes, laughing expression, with talking of religious things, of fulfilling vows, prayers, and the impression that he is elsewhere than at home. Heaviness of the head, during which all things seem to whirl in a circle. Pupils either contracted or dilated. Deafness in one or both ears as if stopped. Bleeding from the nose, or only from the right side, or at night in sleep. Face pale, cold, cadaverous, with sharpened nose and sunken cheeks; blue circles round the eyes; dark red face, also with heat; redness of one cheek and paleness of the other; they are alternately red and pale. Lips dry, black, and cracked. Dryness of the mouth, also with thirst. Tongue red and swollen; dry, blackish, and cracked. Loss of speech, stuttering. Black vomiting, also of black bile and blood, after previous bile and mucus. Great sensitiveness of the abdomen to touch; distention, with hardness; rumbling as if there would be diarrhœa. Nocturnal diarrhœa; copious; with pain during and after the stool; with chill; with tense abdomen; greenish watery with mixed flocks; brown, blackish; unnoticed thin stools when flatus escapes; sense of weakness in the abdomen, like fainting, before the stools; during the stool great weakness, paleness of the face, and cold sweat on the forehead. Involuntary discharge of urine.

The above are the groups of symptoms which the respective drugs produce on the healthy, analogues of which may be met in cases of this fever. It will, of course, be seen at a glance that many of them, perhaps a majority, are like to symptoms only found in the early stage of attacks, when, unfortunately, the patient and his friends still hope for recovery without recourse to professional aid. The abundance of resources here evinced with which to meet the beginnings of the invasion cannot but cause regret that they should be so often suffered to pass into the stage of full development, before they become subjects of professional treatment; and the more because, if, as is too likely to be the case, the patient has been exposed to domestic medication, he has most certainly been damaged. The weariness, debility, dullness of mind, restlessness, want of repose, disturbed sleep, bad dreams, vertigo, headache, pains in the limbs, chilliness, etc., are often not suspected as indicative of any important ailment, still the case has passed to other and graver symptoms, which declare the establishment of a process which is only too likely now to progress against whatever medication, through the other stages of the disease, to recovery or a fatal termination, determined by the intensity of the action of the morbid poison, the constitution of the individual attacked, or the right or wrong employment of remedial agents. That this early stage of the fever is so often allowed to pass without proper attention unquestionably accounts for much of the mortality which results from its unchecked progress. The complete picture of this stage in the symptoms of many of the medicines here given is an assurance to the careful practitioner that, by the timely use of that drug the action of which is most like the symptoms of his case, he may often be able to cut the disease short and secure a convalescence without the suffering or danger which are necessarily met in the course of its full development. Some of the medicines, symptoms of which we have here given, can but rarely be called for in any other stage. This is true of Arnica, and to a considerable extent of Bryonia, Nux vomica, and Pulsatilla. Arnica has but few symptoms like those met in the later stages of the fever, such as loss of consciousness, delirium, bleeding from the nose, distention of the abdomen, and diarrhœa, while there are very many quite common in the initiatory stage. The range of Bryonia is much wider. While it has many symptoms of the early attack, it has also very many common to all stages of the fever. As in other diseases which call for this drug, so in this, the patient seeks entire repose, for all his sufferings are increased by motion. It is never to be overlooked in cases where the lungs are invaded in progress of the fever, especially where there are, with the cough, stitching pains in the chest and pressure on the middle of the sternum, like a weight, with oppressed and anxious respiration. Its place is probably in the early stage of this invasion, before the lung is fully hepatized, after which there is no doubt Phos., Sulph. or Lach. will be found better remedies; which of these is to be determined by the general symptoms of the case. It may be doubted whether Bryonia is ever called for in the last stage of these cases where stupidity has passed into unconsciousness, the evacuations are unnoticed, and there is carphologia, extreme prostration, etc.

*Cocculus* is also especially related to the early stage of the fever, and likewise to that of convalescence. For the relief of the great debility which is often so oppressive, and sometimes protracted, in the convalescent stage, this is often one of the very best remedies. The class of cases to which it is appropriate in either stage is sufficiently obvious after only a cursory attention to the symptoms here given.

*Nux vomica* has many symptoms of the early stage of the fever and also of the second. Its use must be nearly if not entirely limited to these stages. In the early stage, if there be chilliness on the slightest movement, dryness of the front of the mouth and tip of the tongue, intolerance of impressions on the external senses, all of which seem much exaggerated, great sensitiveness to the open air, thirst, with aversion to water, there need be no doubt as to the propriety of giving this remedy or of its beneficial action. It is also characterized by a strong want to lie down and a considerable relief to the sufferer on doing so. In the second stage it is in place, if with these symptoms there be the condition of the mouth and abdomen given above, with the characteristic diarrhœa.

*Phosphorus* has symptoms of the first stage—that of attack—but its great importance is in the second and latter stages, in the treatment of which it often happens there can be found no proper substitute for it. This is especially likely to be the case in the fevers which attack violently both the intestines and the lungs. (See the symptoms of the abdomen, cough, and respiration.) It is invaluable in those cases of congestion of the lungs where the organs are already to a greater or less extent hepatized and are on the eve of degenerating into abscess. In such cases it can give place to no remedy whatever—Lach. or Sulphur may be rather called for, if there be in the solidified lung tissue no disposition to deposit pus. We expect on a future occasion to call attention again to Lachesis as an agent for procuring the absorption of deposits by inflammation into the pulmonary tissue. Its efficacy in such cases is not surpassed even by Sulphur itself.

*Pulsatilla.*—This is appropriate to the stage of attack. It will be noticed the symptoms are marked by intolerance of external heat, by sensation of heat which distresses and causes the rejection of covering, the removal of which is followed by chill; heat of one side, or heat of one side with coldness of the other; perspiration on one side of the face or body; heaviness of the head with intolerance of light. It has also important relations to the later stages and to the severer forms of the fever. This is obvious from the fixed ideas, violent delirium, frightful visions, desire to escape, and from the symptoms of the abdomen and diarrhœa. It has succeeded in rescuing patients from the utmost danger, in these stages, in the experience of the writer, and he feels to recommend it to the careful consideration of his readers in the treatment of cases characterized by the above symptoms.

*Rhus toxicodendron* has been much used in the treatment of this fever, and not always with success, because there has not always been a clear perception of the elements by which it is related to the fever as a curative. When they are wanting its use must be either nugatory or mischievous, and most likely the latter, whatever its success may have been in other cases. There can be no greater folly than giving this or any other drug, in this or any other disease, merely because it is recommended in some of the repertories, or because it has been found specific in some epidemics called, for convenience sake, by the same name. It cannot be too often or too strongly impressed on the mind of the prescriber that the *only* true basis of a prescription is the similarity of the elements of the individual case to the characteristics of the drug. Where this similarity exists there need be no hesitation in the use of the drug in whatever stage of the disease. These remarks are made because it is believed

Rhus has been prescribed in the treatment of this fever out of its place oftener than any other drug, except perhaps Bryonia.

The case of typhoid fever which calls for Rhus is characterized by desire for frequent or constant movement, which seems to give a temporary relief to the patient. The anxiety of Rhus is of the body, and not of body and mind, and is in some degree mitigated by the movements. The delirium is likely to be demonstrative and loquacious, but it lacks the extent of violence which belongs to Belladonna, and also the characteristics of delirium of that drug. Mental operations are difficult and slow, he is stupid. The sleep is restless, disturbed, anxious, with frightful dreams, with frequent waking, and never that of quiet, profound coma. The nearest approach to this is a comatose slumbering, with snoring, murmuring, picking at the bedclothes, and dreams. The patient desires to be covered while sweating; want of memory. Headache when opening the eyes. Bleeding of the nose, *especially after midnight.* The state of the abdomen and diarrhœa are peculiar, and need not be confounded with that which belongs to any other drug. It is not to be forgotten that this remedy is not called for in any case when the patient is comforted by complete and continued repose—in this, as has been remarked, it being the exact opposite of Bryonia.

*Veratrum* is rarely called for in the attacking stage of the fever. It belongs rather to that of great prostration, cold sweating, coma, with a diarrhœa which is quite characteristic. There can hardly be a mistake in its use if the above symptoms are carefully attended to.

